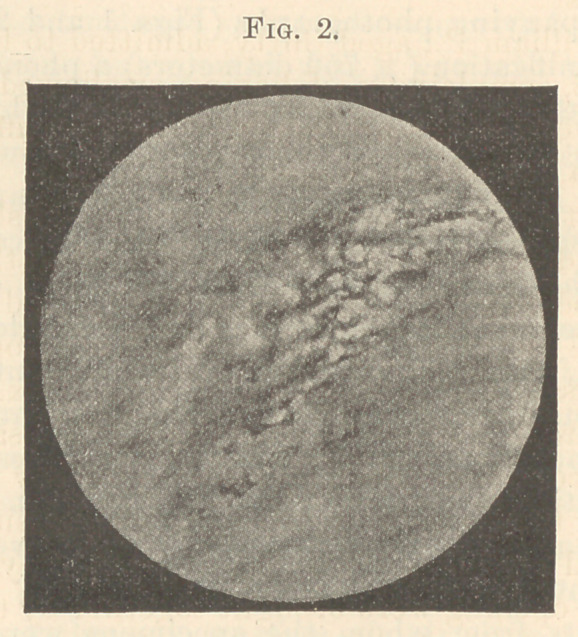# A New Factor in Erosion

**Published:** 1898-10

**Authors:** Arthur S. Underwood


### Abstracts and Translations.

### A NEW FACTOR IN EROSION.[1]

BY ARTHUR S. UNDERWOOD, M.R.C.S., L.D.S.

THE accompanying photographs (Figs. 1 and 2) show under a very high magnification (× 750 diameters) a phenomenon that has never been observed, so far as I am aware, and which throws some light upon the pathology of the form of tooth-wasting which is called erosion. In both sections (Figs. 1 and 2) an unquestionable interglobular space is shown; the calcospherites are extremely minute, but they are calcospherites, and they exist in human enamel. As yet no one has ever shown interglobular spaces in human enamel, although, no doubt, every student of Rainey and Ord who accepts their theories of calcification must suppose that imperfect enamel should contain these appearances.

Neither section was submitted to any reagent in the course of preparation; both were simply ground thin between two slabs of glass and mounted without any stain.

The patients from whom the specimens were obtained were both victims of very extreme and very typical erosion. The shiny grooves, sometimes with sharp edges, ran all over the surfaces of most of the teeth. Here and there caries might be seen running its own course, and in some sections I have stained the micro-organisms with methyl-violet to show the two forms of destruction in marked contrast.

I have never found these spaces except in enamel which was

[1] Being the substance of some remarks made during the discussion on Mr. Storer Bennett's paper on erosion at the Bath meeting.

subject to erosion. I have generally found it scattered through the whole of enamel which was so affected.

Fig. 1.

Fig. 2.

After considerable trouble I obtained, by the kindness of Professor Stewart, of the College of Surgeons, and of Mr. Smith Woodward and Sir W. Flower, some sections of the teeth of the sea-lion, which presented the well-known appearance figured by Dr. Murie in the *Odontological Society's Transactions* (1870). These do not show the spaces,—at least the appearances in the enamel which seem to suggest spaces are very doubtful, and I prefer to leave them out of the argument. Moreover, I do not myself perceive any resemblance to erosion in the change which wastes the teeth of the seal, the latter appearing much more like the result of attrition of a purely mechanical kind, such as might be produced by the rolling of pebbles about in the mouth, a habit attributed to these animals.

I must add that I am indebted to Mr. Andrew Pringle for the perfection with which the sections are reproduced photographically.—*Journal of the British Dental Association.*

## Figures and Tables

**Fig. 1. f1:**
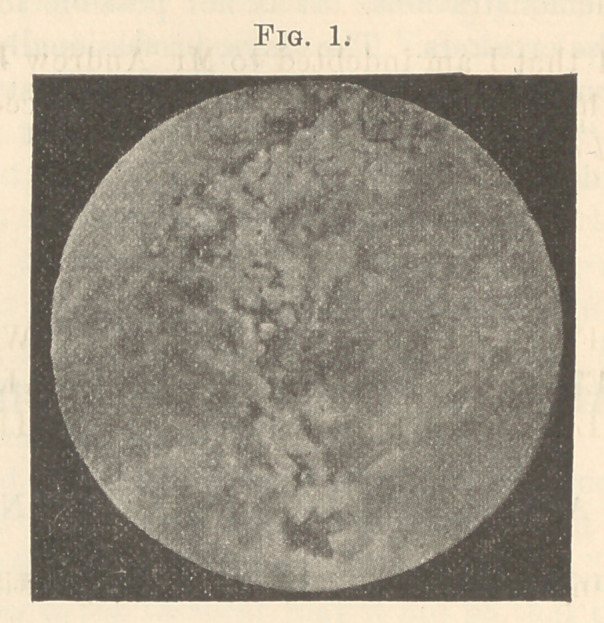


**Fig. 2. f2:**